# Development and optimization of an antibody array method for potential cancer biomarker detection^☆^

**DOI:** 10.1016/S1674-8301(11)60008-0

**Published:** 2011-01

**Authors:** Shuangshuang Wang, Ping Zhao, Brian Cao

**Affiliations:** a Key Laboratory of Antibody Technology of Ministry of Health, Nanjing Medical University, Nanjing, Jiangsu Province 210029, China;; b Antibody Technology Laboratory, Van Andel Research Institute, Grand Rapids, MI 49503, USA.

**Keywords:** cancer biomarker, HPCAL1, PEBP1, LGALS7, SERPINE2, antibody microarray

## Abstract

Biomarkers play an important role in the detection at an early stage of pancreatic cancer. The aim of the present study was to optimize the conditions of antibody arrays for detecting Hippocalcin-like 1 (HPCAL1), phosphatidylethanolamine binding protein 1 (PEBP1), lectin galactoside-binding soluble 7 (LGALS7), and serpin peptidase inhibitor clade E member 2 (SERPINE2) as biomarkers for pancreatic cancer detection in a single assay and to investigate antibodies' specificity and cross-reactivity. Capture antibodies against HPCAL1, PEBP1, LGALS7 and SERPINE2 were printed on nitrocellulose coated glass slides. HPCAL1, PEBP1, LGALS7 and SERPINE2 proteins with different concentrations were incubated with the capture antibodies at different temperatures for different time periods. Biotinylated detection antibodies recognizing a different epitope on the captured proteins and a secondary detection molecule (Streptavidin-PE) were used to detect fluorescent signals. The arrays showed the strongest signals when the concentration of the capture antibodies was at 500 µg/mL in PBST0.05 (PBS with 0.05% Tween-20), and the slides were incubated overnight at 4°C. The lowest protein concentration for detection was 2 ng/mL. Each antibody demonstrated high specificity to the corresponding antigen in detecting a mixture of 4 proteins without significant cross-reactivity. The fluorescence and biomarker concentration displayed a linear correlation. The antibody microarray system could be a useful tool for potential biomarker detection for pancreatic cancer.

## INTRODUCTION

In cancer, a biomarker refers to a substance or process that is indicative of the presence of cancer in the body. Great efforts have been put forth to develop new methods for rapid, sensitive and accurate detection of biomarkers. A biomarker could be either a molecule secreted by tumor or a specific response of the body to the presence of cancer. Genetic, epigenetic, proteomic, glycomic and imaging biomarkers can be used for cancer diagnosis, prognosis and epidemiology study[1]. Biomarkers measured in a variety of patient samples, including blood, tissue, urine and cerebrospinal fluid, are used in a diverse array of clinical settings. The application of biomarkers to cancer is leading the way because of the unique association of genomic changes in cancer cells with the disease process.

Pancreatic cancer is the sixth highest cause of mortality from malignant tumors in Europe and the fourth-highest in the United States[2],[3]. The dismal prognosis of pancreatic cancer is due to the late stage at which it is usually diagnosed because pancreatic cancer patients seldom exhibit disease-specific symptoms until late in the course of the disease[4],[5], and the high invasive and metastatic potential of pancreatic tumors result in a low rate of curative resections and a high frequency of relapse. Long-term survival from pancreatic cancer is possible if the disease is identified at an early stage[6],[7]. Therefore, biomarkers play an important role in the detection at an early stage. Several serum markers have been investigated for pancreatic cancer. Elevated CA19-9 level has been cited as a potential marker of the disease[8]–[12], and other existing biomarkers relate to the inflammation that associates with the tumor and other pancreatic diseases that may be present[13]–[15].

Recently, various microarray formats have been utilized for studying glycosylation patterns[16],[17]. In one study examining serum samples from patients with colon and pancreatic cancers, glycoproteins extracted from the serum were printed on glass slides and hybridized against various lectins to study changes in the glycan patterns during cancer progression[18],[19]. The antibody microarray is a favorable format for high throughput analysis, with high specificity and reproducibility[20]–[22].

In 2007, the Lustgarten Foundation undertook an extensive bioinformatics analysis of the published literature to identify the top 60 most promising biomarkers for pancreatic cancer. In the present study, we selected four biomarkers Hippocalcin-like 1 (HPCAL1), phosphatidylethanolamine binding protein 1 (PEBP1), lectin galactoside-binding soluble 7 (LGALS7) and serpin peptidase inhibitor clade E member 2 (SERPINE2) to develop an antibody microarray system that could have potential clinical diagnostic applications. The current study focused on two areas: one was to optimize the standard curves of these four biomarkers so that they can be used to calculate the concentration of biomarkers quantitatively; the other was to investigate the cross-reactivity and to further develop array-based assay so that multiple biomarkers can be detected in a single assay.

## MATERIALS AND METHODS

### Materials

HPCAL1, PEBP1, LGALS7 and SERPINE2 proteins and corresponding antibodies were generated as previously described[23]. Antibody microarrays were prepared as previously described[24].

### Methods

**Fig. 1:**
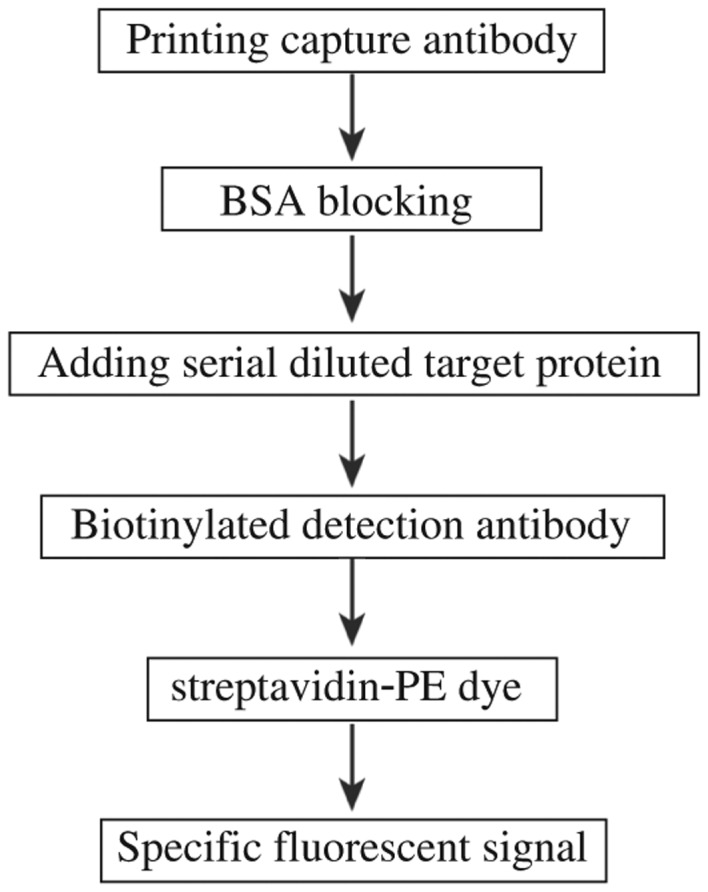
An outline of the experiment flow of microarray processing.

**Fig. 2:**
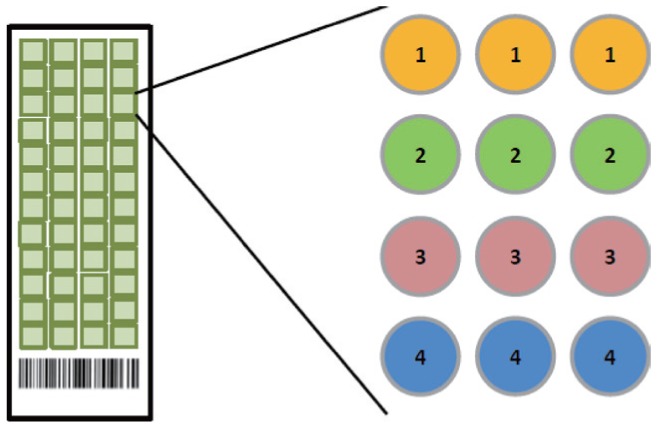
Microarray slides. The microarray was patterned as follows: 1) the first row with anti-HPCAL1 (5A5), diluted in PBST0.05; 2) the second row with anti-HPCAL1 (5A5), diluted in PBS; 3) the third row with anti-PEBP1 (8E2), diluted in PBST0.05; and 4) the last row with anti-PEBP1 (8E2), diluted in PBS. The antibody concentration was 250 µg/mL.

An outline of the experiment flow of microarray proecessing is described in ***Fig 1***. A piezoelectric contact printer (Auson 2470 Arrayer) was used to spot approximately 260 pL of each antibody solution at a certain concentration on the surfaces of nitrocellulose-coated glass microscope slides (PATH slides; GenTel Biosciences, Madison, USA). Forty-eight identical arrays were printed on each slide, with each array consisting of two different antibodies at first. When the conditions were optimized, then coated four antibodies were coated to detect the cross-reactivity. All of the antibodies were mouse monoclonal antibodies targeting human proteins HPCAL1, PEBP1, LGALS7 and SERPINE2. Each capture antibody was printed in triplicate, and biotinylated BSA was printed as control (***Fig. 2***). A wax border was imprinted around each of the arrays to define hydrophobic boundaries (SlideImprinter, The Gel Company, San Francisco, CA, USA). The printed slides were stored at 4°C in a desiccated, vacuum-sealed slide box until use.

For condition optimization, capture antibodies printed on slides were diluted in two different buffers: PBS and PBST0.05 (PBS with 0.05% Tween-20), and with three different concentrations: 250 µg/mL, 500 µg/mL, and 750 µg/mL. For multi-marker detection system, capture antibodies were only diluted in PBST0.05 with one concentration at 500 µg/mL. First, vacuum sealed arrays were equilibrated with ambient temperature for 30 min before use. Slides were washed in PBST0.5 (PBS with 0.5% Tween-20) for 3 min with gentle shaking, and dried by centrifuging for 2 min at 900 *g*. Then, the slides were blocked with 1% BSA in PBST0.5 for 1 h in a humidified chamber to prevent from evaporating. Excess capture antibodies were washed away with the blocking buffer by shaking. A serial dilution of protein or protein mixture samples containing 0.1% Brij-35 (Pierce, Rockford, IL, USA) in PBST0.1 (PBS with 0.1% Tween-20) were applied and incubated for 1 h at room temperature (RT) or overnight at 4°C with gentle agitation. Excess protein was removed by three washes with PBST0.5, each for 3 min with shaking. Then biotinylated detection antibodies either along or mixed together, were diluted to 3 µg/mL with 0.1% BSA/PBST0.1 and added to the corresponding blocks, incubating for 1 h at RT. The arrays were washed with PBST0.5 and blocked briefly with 1% BSA/PBS before probing with streptavidin-PE. The streptavidin-PE was diluted to 2 µg/mL with PBST containing 0.1% BSA and probed to the array for 1 h. The labeled slide was washed with PBST0.5 for three times, and dried by centrifugation. The slides were scanned using a GenePix 4000B microarray scanner, and the data were processed using GenePix Pro 3.0 software[25].

## RESULTS

### Comparison of capture antibodies diluted in PBS and PBST0.05

**Fig. 3:**
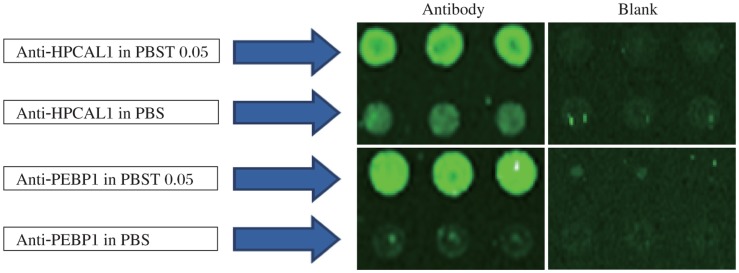
Comparison of capture antibodies diluted in PBS and PBST0.05. Microarray slides were patterned as in ***Fig 1***. The two proteins were applied to the arrays, respectively, followed by detecting with detection antibodies and fluorescence labeling with PE.

Using the microarray format, we compared the conditions of the experiment. First, we printed two capture antibodies anti-HPCAL1 5A5 and anti-PEBP1 8E2 in two different buffers, PBS and PBS with 0.05% Tween-20. After blocking and washing away the excess antibodies, serially diluted HPCAL1 and PEBP1 proteins were applied to the multiplexed arrays. Following incubation and washes, the arrays were probed with the respective biotinylated detection antibodies anti-HPCAL1 1E10 and anti-PEBP1 4F10. Fluorescent signal was generated by incubating the arrays with streptavidin-PE. The experiment was repeated for confirmation. The results in ***Fig. 3*** showed that antibodies in PBST0.05 had much stronger signals than in PBS.

### Concentration comparison of capture antibodies

**Fig. 4:**
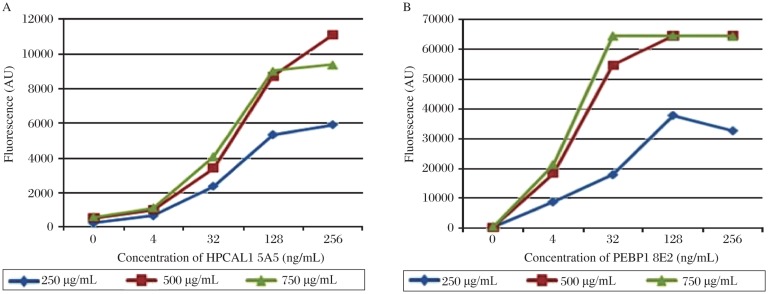
Concentration comparison of the capture antibodies. (A) Serially diluted HPCAL1 in PBST0.1 containing 0.1% Brij were applied to the arrays which were printed with different concentrations of the capture antibody anti-HPCAL1 5A5. (B) Serially diluted PEBP1 in PBST0.1 containing 0.1% Brij were applied to the arrays which were printed with different concentrations of the capture antibody anti-PEBP1 8E2.

To optimize the concentrations of the capture antibodies, we printed slides with 3 different concentrations of the two capture antibodies, anti-HPCAL1 5A5 and anti-PEBP1 8E2 at 250 µg/mL, 500 µg/mL and 750 µg/mL. Serially diluted HPCAL1 and PEBP1 were applied to the arrays, respectively. The results showed, that the concentrations of 500 µg/mL and 750 µg/mL had no significant impact on fluorescent signal; signals might be saturated at the concentration of 750 µg/mL. So the optimal concentration for the capture antibodies was set to 500 µg/mL (***Fig. 4***).

### Incubation temperature comparison

**Fig. 5:**
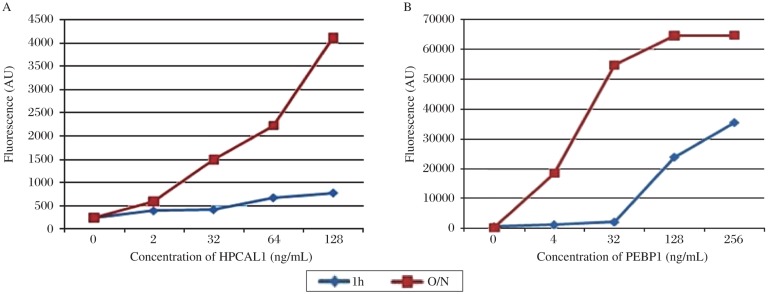
Comparison of protein incubation for 1 h at room temperature and overnight at 4°C. The results showed that the signal of overnight at incubation 4°C was much stronger than that of 1 h at room temperature. A: HPCAL1; B: PEBP1.

To optimize the incubation temperature conditions of the antibody array, after HPCAL1 and PEBP1 proteins were applied to the arrays, the slides were incubated either overnight (O/N) at 4°C, or for 1 h at RT separately with the same other conditions. The results showed that the signal of overnight incubation at 4°C is much stronger than 1 h at RT (***Fig. 5***).

### Antibody specificity and cross-reactivity test

**Fig. 6:**
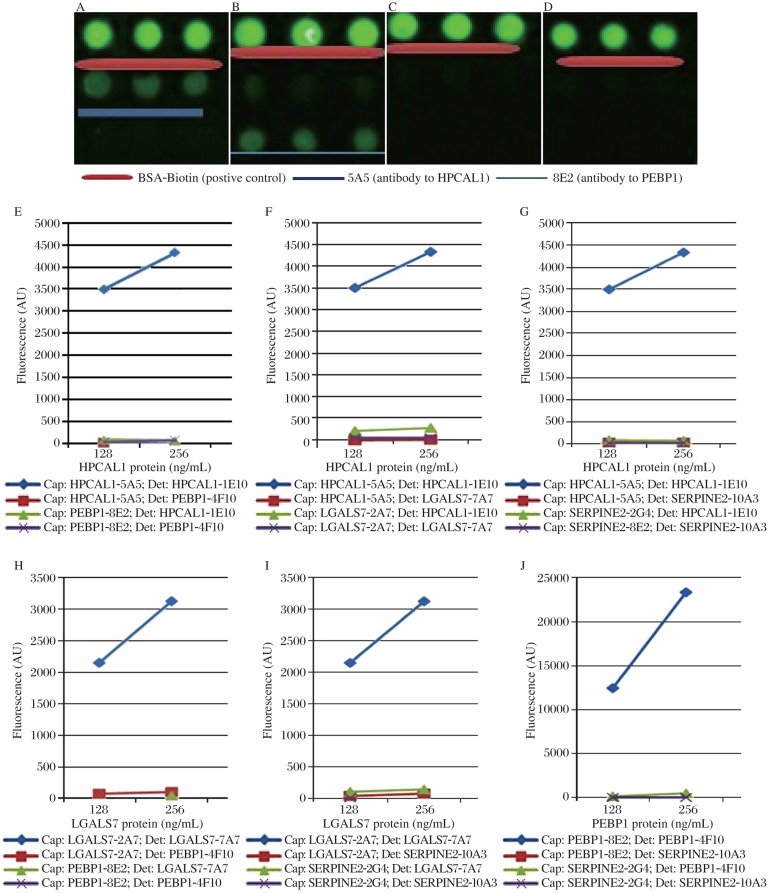
Antibody specificity tests. Firstly, the cross-reactivity was tested between HPCAL1 and PEBP1, and the results (A-E) showed that there was no cross-reactivity observed. A: HPCAL1 protein was added and the detection antibody was HPCAL1 1E10. B: PEBP1 protein was added and the detection was PEBP1 4F10. C: HPCAL1 protein was added, and the detection antibody was PEBP1 4F10. D: PEBP1 protein was added, and the detection antibody was HPCAL1 1E10. Secondly, the cross-reactivity tests were conducted among HPCAL1-5A5, PEBP1-8E2, LGALS7-2A7 and SERPINE2-2G4. The results showed that there were no cross-reactivities betweeh each two of the four proteins. E: cross-reactivity tests between HPCAL1 and PEBP1. F: cross-reactivity tests between HPCAL1 and LGALS7. G: cross-reactivity tests between HPCAL1 and SERPINE2. H: cross-reactivity tests between LGALS7 and PEBP1. I: cross-reactivity tests between LGALS7 and SERPINE2. J: cross-reactivity test between PEBP1 and SERPINE2. Cap: Capture antibody; Det: Detection antibody.

After the conditions were optimized (the capture antibody at 500 ng/mL in PBST0.05, and incubation overnight at 4°C), we next investigated the specificities of each antibody, and whether there were cross-reactivity among antibodies to different proteins. In this experiment, four sets of capture and detection antibodies against four different antigens (HPCAL1, PEBP1, LGALS7 and SERPINE2) were compared and analyzed in all possible combinations. As shown in ***Fig. 6***, for example, when HPCAL1 protein was present, HPCAL1 capture Ab 5A5 only captured HPCAL1, and its detection Ab 1E10 only detected HPCAL1 protein. These results demonstrated that each capture and detection antibody were specific to their corresponding protein, and the cross-reactivity to other proteins was either none or extremely low. The protein concentrations were set to high at 125 ng/mL or 256 ng/mL to avoid background interference.

### Simultaneous detection of HPCAL1, PEBP1, LGALS7 and SERPINE2

**Fig. 7:**
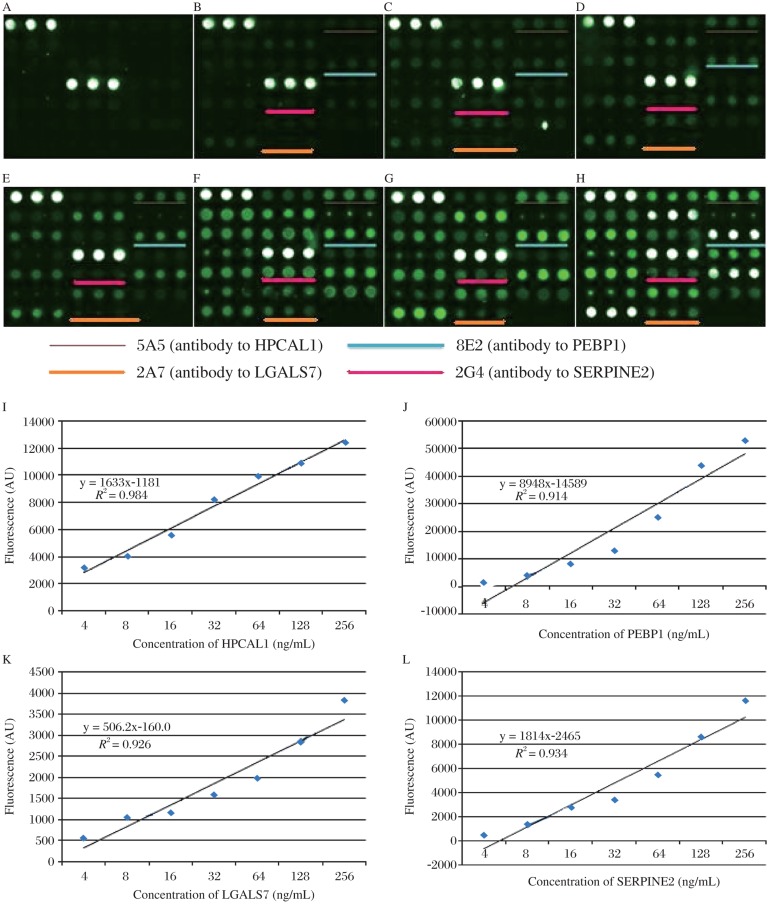
Simultaneous detection of HPCAL1, PEBP1, LGALS7 and SERPINE2 by detection cocktail antibodies. Serially diluted mixture of HPCAL1, PEBP1, LGALS7 and SERPINE2 (A: blank control. B: 2 ng/mL. C: 4 ng/mL. D: 16 ng/mL. E: 32 ng/mL. F: 64 ng/mL. G: 128 ng/mL. H: 256 ng/mL) were probed, followed by detection with antibody cocktail. The other spots not underlined were other different proteins not shown in this paper. I-L showed that when the concentration of these four markers were below 256 ng/mL, the fluorescence and biomarker concentration displayed a linear correlation (I: HPCAL. J: PEBP1. K: LGALS7. L: SERPINE2).

Our final goal was to establish an assay system which allowed detecting multiple biomarkers in a single setting. We proved that the antibodies to each protein were specific and did not cross-react with any other proteins (***Fig. 6***). In this experiment, capture antibodies were printed onto slide separately; four different proteins were mixed and incubated with capture antibodies on the slide followed by incubating with mixed detection antibodies. The results in ***Fig. 7(A-H)*** showed that the sensitivity reached to 2 ng/ml. When the concentrations of these four biomarkers were below 256 ng/ml, the fluorescence and biomarker concentration displayed a liner correlation shown in ***Fig. 7(I-L)***.

## DISCUSSION

In this study we optimized the conditions of antibody arrays. Four potential biomarkers for pancreatic cancer diagnosis were chosen, and their corresponding antibodies were printed on coated glass slides. We found that when the concentration of the capture antibodies was at 500 µg/ml in PBST0.05 and the slides were incubated overnight at 4°C, the arrays gave the strongest signals. Simultaneous detection of these four markers worked very well with almost no cross-reactivity. Moreover, the fluorescence and biomarker concentration displayed a linear correlation.

The experimental features of microarrays have advantages for cancer research. The advantage of low sample volume results in the consumption of small amounts of both precious clinical samples and expensive antibodies. The assays can be run efficiently in parallel, enabling studies on large populations of samples that are necessary for biomarker discovery and validation. In addition, the assays have good reproducibility, high sensitivity and quantitative accuracy over large concentration ranges[26].

In the future, we hope to establish collaboration with hospitals or clinical diagnostic laboratories, run pancreatic cancer serum samples using antibody array format to identify differences in these potential biomarkers compared to normal and pancreatitis serum samples. Patient sera with different types and stages of pancreatic cancer will also be examined. Pancreatic cancer continues to have a high mortality rate due to detection at a late stage of the disease. We hope that the antibody array technology would eventually be able to provide a practical means to characterize patterns of variation in hundreds of thousands of different proteins in clinic and research applications.
